# Current Diagnosis and Management of Ocular Graft-Versus-Host Disease at a Tertiary Cancer Center

**DOI:** 10.3390/jcm15051926

**Published:** 2026-03-03

**Authors:** Eesa M. Khattak, Nathan A. Seto, Calvin W. Wong, Rugveda R. Patil, Dan S. Gombos, Joshua L. Olson, Richard W. Yee

**Affiliations:** 1 School of Medicine, Royal College of Surgeons in Ireland-Bahrain, Muharraq 15503, Bahrain; 2 Richard W. Yee, MD PLLC, Houston, TX 77401, USA; 3 Department of Ophthalmology, McGovern Medical School, The University of Texas Health Science Center at Houston, Houston, TX 77030, USA; 4 Department of Biology, Texas A and M University, College Station, TX 77843, USA; 5 Department of Head and Neck Surgery, The University of Texas MD Anderson Cancer Center, Houston, TX 77030, USA; 6 School of Medicine, University of Limerick, V94 T9PX Limerick, Ireland

**Keywords:** ocular graft-versus-host disease, allogeneic hematopoietic stem cell transplant, chronic graft-versus-host disease, dry eye disease, ocular surface disease, tissue-based therapy

## Abstract

Ocular graft-versus-host disease (oGVHD) remains one of the most challenging complications of allogeneic hematopoietic stem cell transplantation (HSCT), often leading to severe ocular surface morbidity and irreversible vision loss if not properly managed. Diagnostic uncertainty persists due to variability in clinical presentation and a lack of universally accepted criteria, but the greatest clinical burden lies in establishing effective, durable treatment protocols. Current strategies range from lubricants and topical immunomodulators to advanced surgical interventions; however, outcomes remain inconsistent due to delayed recognition and heterogeneous practices across institutions. At institutions like MD Anderson Cancer Center (MDACC), a structured treatment strategy has been developed that emphasizes early recognition and targeted therapy based on the specific ocular tissues affected. This approach integrates patient-reported outcomes with objective ocular findings and applies stepwise therapeutic escalation aligned with tissue-specific pathology. This review offers a brief overview of the clinical burden and pathophysiology of oGVHD, outlines the key diagnostic challenges, and a more detailed discussion on therapeutic strategies with particular emphasis on the targeted tissue-based approaches.

## 1. Overview of Graft-Versus-Host Disease

GVHD is driven by donor T cells. However, the specific antigens, cellular subsets involved, and underlying mechanisms of chronic GVHD remain incompletely understood and may vary [1,2]. GVHD is traditionally classified into two clinical entities: acute GVHD (aGVHD) and chronic GVHD (cGVHD). aGVHD typically develops within the first three months following transplantation and commonly affects the skin, gastrointestinal tract, and liver [3]. aGVHD develops in approximately 40–60% of recipients of HLA-matched sibling grafts and in up to 80% of those receiving unrelated donor grafts [4]. In contrast, cGVHD arises from persistent immune dysregulation involving both T and B lymphocytes and is characterized by chronic inflammation, aberrant tissue repair, and progressive fibrosis across multiple organ systems [5]. The estimated incidence of chronic GVHD ranges from 30% to 70% among allo-HSCT recipients who survive beyond 100 days post-transplant [6]. Notably, cGVHD profoundly increases morbidity, increases mortality, and reduces patients’ quality of life (QoL) [7].

Among the organ manifestations of cGVHD, ocular involvement is one of the most common and functionally debilitating, requiring dedicated attention in post-transplant care [1].

### Ocular Involvement in GVHD

Ocular involvement is among the most frequent organ manifestations of cGVHD and is reported in 30% to 85% of affected individuals. oGVHD is mediated by donor T cell recognition of ocular tissues as foreign. This immune recognition initiates a T cell–mediated inflammatory cascade, resulting in chronic damage to ocular surface structures and persistent symptoms [8]. Clinically, patients commonly present with dryness, ocular irritation, conjunctival redness, intermittent blurring of vision, photophobia, and ocular pain [9]. Reduced scores in domains such as general vision, ocular pain, vision-specific mental health, and vision-specific role difficulties indicate significant burden on QoL [10]. oGVHD is associated with a relatively poor prognosis, closely reflecting the overall severity and progression of the disease. For example, patients with severe oGVHD demonstrate a significantly lower 5-year survival rate compared to those with non-severe forms [11].

## 2. Pathophysiology of oGVHD

oGVHD occurs due to donor-derived immune-mediated injury to multiple components of the ocular surface system. It develops when donor-derived T cells recognize host ocular surface tissues as foreign, triggering a persistent immune response. This immune reaction begins with T-cell infiltration of target tissues, where activated T cells release pro-inflammatory cytokines such as IL-1, TNF-α, and IFN-γ [1]. These mediators drive chronic inflammation and immune cell recruitment. Over time, this sustained inflammation promotes fibroblast activation and extracellular matrix deposition, leading to progressive fibrosis of lacrimal and meibomian glands. The resulting glandular dysfunction impairs tear secretion and tear film stability, ultimately causing persistent epithelial damage and ocular surface disease in oGVHD [12].

The clinical manifestations and objective findings of the disease vary according to the specific tissues involved. oGVHD classically affects the lacrimal glands, meibomian glands, conjunctiva, and cornea. Each affected site presents with characteristic signs, symptoms, and functional impairments, leading to a diverse range of presentations across patients.

### 2.1. Lacrimal Gland Dysfunction

Lacrimal gland dysfunction (LGD) is one of the most common ocular manifestations of oGVHD [13]. While the exact mechanism remains partially understood, LGD is generally attributed to immune-mediated injury and secondary fibrotic changes. Donor-derived T-cell infiltration and subsequent pro-inflammatory cytokine release damage lacrimal acinar cells, while fibrotic remodeling of the conjunctiva impairs the function of accessory lacrimal glands [1]. Fibrotic changes to the conjunctiva also disrupt meibomian gland lipid delivery to the tear film, which increases evaporation of the tear layer. Early immunohistochemical studies of oGVHD-affected lacrimal glands identified the presence of activated CD34^+^ fibroblasts, indicating fibroblast-driven fibrosis as a key factor in glandular dysfunction [14]. Recent studies suggest renin-angiotensin system (RAS) activation plays a role in oGVHD lacrimal gland fibrosis, with the angiotensin II type 1 receptor (AT1R) blockade shown to reduce inflammation and improve tear production in experimental models [1]. The resulting aqueous tear deficiency contributes to tear film instability and amplifies ocular surface inflammation.

### 2.2. Conjunctival Involvement

Conjunctival involvement is less common in both acute and chronic GVHD. However, when present, it is often a poor prognostic indicator and may reflect systemic disease dissemination [13,15,16]. Classification schemes for conjunctival involvement in GVHD vary. Milder cases typically present with conjunctival hyperemia and mild erythema, while more severe cases may manifest as pseudomembranous conjunctivitis, cicatrizing conjunctivitis, and other advanced ocular surface pathologies [1,16]. Histologically, conjunctival involvement in GVHD is characterized by a reduction in goblet cell density and squamous metaplasia, and increased surface keratinization of the ocular epithelium [17]. Collectively, these alterations disrupt tear film stability, enhance chronic ocular surface inflammation, and, in advanced cases, may lead to irreversible vision loss [18].

### 2.3. Meibomian Gland Dysfunction

Meibomian gland dysfunction (MGD) is a common feature of oGVHD, affecting roughly 47% to 80% of oGVHD patients [1]. The meibomian glands, located along the eyelid margins, secrete meibum, an oily substance which forms the outer layer of the tear film and slows evaporation. In oGVHD, these glands can become blocked due to hyperkeratinization, vascular changes, or displacement of the mucocutaneous junction. The quality and quantity of meibum are often reduced, and many glands shrink or disappear [1,16]. Imaging and tissue studies have shown immune cell infiltration, destruction of the oil-producing acinar cells, and fibrosis in MGD. Meibomian gland loss is an early and progressive feature of oGVHD, and it is thought that MGD in oGVHD is due to a disease-specific pathological mechanism rather than nonspecific evaporative dry eye [19]. The resulting loss of oil component destabilizes the tear film, causing excessive evaporation and exacerbating ocular surface inflammation in proportion to the severity of oGVHD.

### 2.4. Corneal Dysfunction

Corneal involvement is a significant manifestation of oGVHD that arises secondary to the inflammation and fibrosis of the lacrimal glands, meibomian glands, and the eyelids [20]. More commonly seen in cGVHD rather than aGVHD, corneal impairment is directly correlated with decreased vision [13]. The common association of cGVHD with keratoconjunctivitis sicca (KCS) leads to tear film instability and aqueous deficiency, which impair epithelial healing and promote punctate epithelial erosions that may progress to persistent epithelial defects (PEDs) [13]. Chronic immune-mediated inflammation leads to damage of the corneal epithelium, basement membrane, and stromal layers due to persistent exposure to inflammatory cytokines and immune cell infiltration [13,21]. Inflammatory mediators include infiltrating donor-derived T cells, activated macrophages, proinflammatory cytokines and proteolytic enzymes such as matrix metalloproteinases (MMPs) [20]. Additionally, progressive corneal nerve damage has been observed in oGVHD patients, resulting in reduced corneal sensitivity and neurotrophic keratopathy [22]. These pathological changes increase the risk of sight-threatening complications such as corneal ulceration, neovascularization, and in severe cases, corneal perforation [20].

## 3. Clinical Presentation and Diagnostic Challenges of Ocular GVHD

### 3.1. Clinical Symptoms and Signs

Among patients who initially present with aGVHD, the first sign of ocular involvement is often the onset of excessive tearing with or without ocular pain, mucous discharge, or morning crusting in the absence of significant irritation [16]. However, these features can be mistaken for infectious conjunctivitis, resulting in unnecessary antibiotic use [23]. The classic symptoms of oGVHD are dryness, excessive tearing, ocular irritation, foreign body sensation (FBS), red eye, intermittent blurring of vision, photophobia, and ocular pain [9,24]. Symptoms vary between patients but often occur in recognizable clusters alongside corresponding clinical signs.

Objective signs are critical in the diagnosis of oGVHD, as symptom–sign discordance is well recognized, and patients’ reported symptoms often do not align with clinical findings. Objective signs crucial for diagnosing oGVHD include slit-lamp evidence of conjunctival scarring, meibomian gland dysfunction, and eyelid margin keratinization. Appropriate assessments of tear function include tests such as Schirmer’s 1, tear breakup time (TBUT), and osmolarity. The application of fluorescein or lissamine green staining to the eye is used to assess epithelial damage and keratopathy. A comprehensive ophthalmologic examination incorporating these findings is essential for accurate identification and monitoring of oGVHD [25].

Other ocular manifestations can provide supportive diagnostic evidence. Eyelid abnormalities such as lagophthalmos, trichiasis, poliosis, entropion, and ectropion arise from cicatricial changes and chronic inflammation [16]. Ocular hypertension and glaucoma have also been documented, with one study reporting ocular hypertension secondary to steroid use in 33 of 218 patients with oGVHD [1]. Other complications include cataract formation, often linked to chronic or repeat glucocorticoid use, and inflammatory conditions such as episcleritis, scleritis, posterior scleritis, anterior uveitis, vitritis, and, rarely, serous choroidal detachment [16]. These findings highlight the broad spectrum of objective signs that extend beyond dry eye, emphasizing the utmost importance for a thorough ophthalmologic evaluation in oGVHD. Whenever possible, a complete baseline ocular surface symptom profile such as the OSDI and complete ocular exam is essential prior to any stem cell transplant. Many times, oGVHD comorbidities (i.e., loss of meibomian glands and abnormal Schirmer’s 1) were present even prior to the cancer diagnosis or presented because of chemotherapies prior to stem cell transplantation.

### 3.2. Symptom-Sign Discordance

The diagnosis of oGVHD remains challenging due to the considerable overlap with other ocular surface diseases, variability in clinical presentation, and the lack of universally accepted diagnostic criteria [26]. For example, patients with marked ocular surface staining and tear deficiency may remain asymptomatic, while others report significant discomfort despite only mild objective findings. In a study of dry eye patients, those with oGVHD were found to demonstrate disproportionately severe ocular surface signs despite reporting fewer symptoms, indicating a significant disconnect between clinical findings and patient perception [27]. This mismatch between signs and symptoms carries important clinical consequences, as reliance on symptoms alone risks delayed diagnosis and underestimation of disease severity. Accordingly, comprehensive evaluation incorporating objective measures such as Schirmer’s 1 testing, corneal fluorescein staining (CFS), and meibography is essential, and has been emphasized in composite diagnostic systems like the National Institutes of Health (NIH) and International Chronic Ocular Graft-Versus-Host Disease Consensus Group (ICCGVHD) criteria [25].

### 3.3. Mimicry of Other Diseases & Misdiagnosis

Another important diagnostic challenge in oGVHD is its ability to mimic other diseases. One of the main mimickers of oGVHD is Sjögren’s syndrome, as both present with aqueous tear deficiency, conjunctival inflammation, and similar dry eye symptoms. The key difference is that Sjögren’s syndrome results from autoimmune exocrinopathy, whereas oGVHD is transplant-related and often more fibrosis-predominant [28]. Conversely, oGVHD can also resemble non-Sjögren’s dry eye disease, as both may present with markedly reduced Schirmer’s 1 scores (<5 mm) in the absence of systemic autoimmunity. Patients with non-Sjögren’s dry eye may also exhibit concurrent meibomian gland dysfunction, further complicating differentiation between the two conditions [28]. In the absence of transplant history and systemic context, distinguishing oGVHD from other forms of dry eye disease can become nearly impossible. In other circumstances, patients presenting with allergic conditions such as allergic conjunctivitis may exhibit redness, itching, and ocular irritation, which can easily be mistaken for symptoms of oGVHD. This tendency toward misdiagnosis often contributes to significant delays in recognition and treatment, with one study reporting that 70% of oGVHD patients were not diagnosed until more than three months after symptom onset, and that 76% were only identified once the disease had already progressed to a severe stage [29].

### 3.4. Lack of Standardized Diagnostic Criteria

A major issue in the diagnosis of oGVHD is the lack of standardized criteria. The existence of different co-existing systems such as the NIH, ICCGVHD, and the Tear Film & Ocular Surface Society Dry Eye Workshop II (TFOS DEWS II), highlight the lack of one single universally accepted standard care. This variability shows the difficulty in establishing a consistent and reliable diagnosis across studies and clinical settings [30].

The NIH guidelines primarily emphasize the dry eye component of oGVHD, relying on patient-reported symptoms, Schirmer’s 1 testing, and the need for lubricant eye drops to relieve symptoms, while not requiring slit-lamp examination [31]. This narrow approach has been criticized for its limited sensitivity and tendency to underdiagnose, making the criteria controversial in clinical practice. Despite these shortcomings, the NIH criteria remain widely used by hematologists, primarily because the criteria can be applied easily without specialized ophthalmologic equipment or expertise. Nonetheless, the NIH criteria’s reliance on subjective and behavior-related measures, while excluding key ophthalmologic assessments such as slit-lamp evaluation and additional ocular surface tests, continues to raise concern about their diagnostic accuracy, especially given the mentioned symptom-sign discordance in oGVHD [30].

In contrast, the ICCGVHD criteria offer a more balanced approach by incorporating both patient-reported symptoms and objective clinical findings. The scoring system includes the Schirmer’s 1 test, corneal fluorescein staining (CFS), conjunctival injection, and the Ocular Surface Disease Index (OSDI), with the final score is also influenced by the presence of systemic GVHD. ICCGVHD guidelines are generally preferred in clinical and hospital settings because they integrate subjective symptoms with measurable ocular signs, providing a more comprehensive and reliable framework for diagnosis [25,30].

The TFOS DEWS II criteria overlap with ICCGVHD in incorporating both symptoms via OSDI questionnaire, and objective signs, but expand the assessment by including additional specialized ophthalmologic tests such as tear film breakup time (TFBUT), tear osmolarity, and detailed ocular surface staining with fluorescein [32]. While these tests provide helpful adjunctive information on tear film function in oGVHD, adoption may only be possible in a narrow range of clinical settings given the requirement of specialized equipment, personnel, and additional time.

Taken together, the coexistence of multiple diagnostic frameworks highlights the lack of consistency across criteria, which in turn shows the need for standardized, universally accepted guidelines. Although each of these guidelines offers valuable insights, their respective weaknesses contribute to delayed recognition and misdiagnosis of oGVHD, which in turn postpones appropriate treatment or results in suboptimal management, ultimately worsening disease progression and compromising patients’ QoL.

## 4. Management of Ocular GVHD & MDACC Institutional Approach

### 4.1. Lack of Standardized Care

The management of ocular graft-versus-host disease (oGVHD) continues to lack universally accepted treatment guidelines, and practice varies widely across institutions. Despite decades of investigation, only six randomized phase III trials have ever been conducted for initial treatment of chronic GVHD, with inconsistent results and no clear consensus on optimal therapy [33]. Reviews of secondary treatment studies similarly reveal major methodological deficiencies, showing that fewer than 10% of reports used consistent regimens, minimized patient selection bias, or applied formal statistical hypotheses, making comparisons across studies unreliable [33,34,35]. In oGVHD specifically, the lack of universally accepted treatment guidelines is further complicated by the existence of multiple diagnostic frameworks (NIH, ICCGVHD, DEWS II), each relying on different combinations of symptoms and objective signs, which alters uniform treatment decisions [26]. Clinical outcomes are still variable, as therapeutic choices often depend on physician preference rather than standardized algorithms, with studies showing that interventions ranging from lubricants to advanced surgical procedures are applied inconsistently across centers [26]. Collectively, these findings demonstrate the need for established, evidence-based, universally accepted treatment guidelines that can optimize patient care and lead to improved clinical outcomes.

### 4.2. Diagnostic and Clinical Evaluation at MDACC

#### 4.2.1. Symptom Evaluation

At MDACC, the management of patients with oGVHD begins with a thorough assessment of patient-reported symptoms. Patients may present with ocular complaints such as discomfort, irritation, pain, dryness, visual disturbance, or foreign body sensation (FBS). Because these symptoms are inherently subjective, a quantifiable global symptom score (GS) is obtained. Patients are asked to rate the severity of their symptoms on a 0–10 scale, where 0 represents no symptoms and 10 indicates the most severe manifestation. This symptom scoring system enables clinicians to capture the patient’s perceived disease burden and track changes over time. Establishment of a GS, especially at a baseline examination, is important for both clinician and patient. When tracked sequentially over multiple visits, trends in symptomatic changes can be easily compared and adjustments to treatment plans can be made depending on the trend. For instance, if a patient’s global score does not improve after initiating therapy, this may indicate the need for reevaluation of the objective ocular surface exam and possible treatment revaluation or treatment escalation. This is performed with careful evaluation of patient compliance and adjusting medication doses, altering therapeutic agents, or modifying treatment topical or systemic oGVHD treatment regimens.

At institutions like MDACC, diagnosis of oGVHD is guided by the ICCGVHD criteria. Patients are given an OSDI questionnaire prior to their exam, and the resulting score is incorporated into the overall calculation to determine the probability and severity of oGVHD. In addition, physicians must be informed whether the patient’s GVHD is systemically active or quiescent, as this significantly influences the final diagnostic classification and severity assessment.

**Figure 1:**
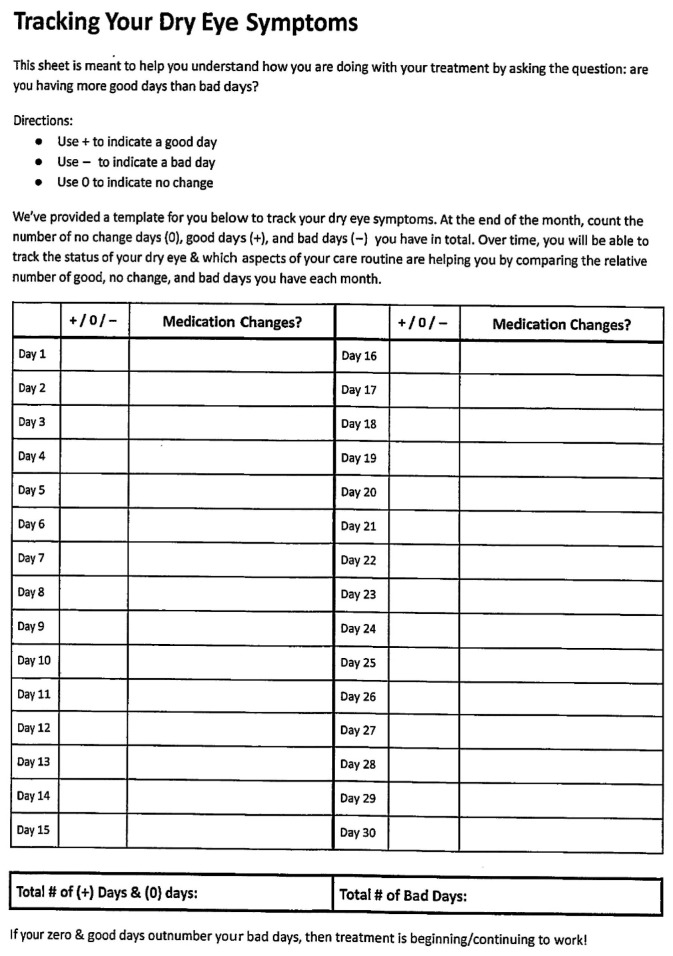
Example of a symptom tracking sheet that is given to patients to assess changes in symptomatology during their treatment.

Furthermore, patients are also provided with a symptom tracking sheet to help both clinicians and patients evaluate the therapeutic benefit of prescribed medications, treatment routines or procedures (Figure 1).

The tracking sheet is structured as a daily log with columns to record whether a medication, treatment routine or ocular surface procedure, when added or removed, affects the patient’s symptomatology over an extended period.

For example, when a patient starts a new topical agent, they will begin recording their subjective impression of how the eye feels on each given day with the new topical agent. The patient’s overall day response will be one of the following: “+” for improving symptoms, “0” for no change, and “−” for worsening symptoms. At the end of the tracking period, usually over a 2-week interval, the total number of “+”, “0”, and “−” days are reviewed. If positive and neutral days outnumber negative days, the medication change is considered beneficial; conversely, if negative days predominate, the added topical agent is considered possibly not beneficial, and an adjustment is reconsidered.

The tracking sheet enables clinicians to correlate symptom patterns with medication changes, providing clearer insight into which treatments are effective, and which may need to be modified if starting or stopping treatment. Whenever possible, emphasize treating the ocular surface with one agent at a time to prevent multiple treatment confusion during the assessment of the ocular surface.

In addition to symptom evaluation, it is standard practice at MDACC to review the patient’s current medications at every visit. Constant evaluation and monitoring of a patient’s medication regimen ensure accurate documentation of therapies already prescribed and help identify potential issues such as non-adherence, incorrect dosing, or confusion between medications. Since these factors can plausibly explain a lack of clinical improvement, a comprehensive medication check is considered compulsory before proceeding with examination or prescribing new treatment.

#### 4.2.2. ICCGVHD Diagnostic Criteria

Once the patient’s symptoms and medication history have been reviewed and the OSDI completed, the next step is a comprehensive ophthalmic examination that incorporates the necessary components of the ICCGVHD criteria along with a general evaluation performed for every patient.

Under the ICCGVHD criteria, the objective measures used to establish both diagnosis and severity include the Schirmer’s 1 test, conjunctival injection, and corneal fluorescein staining (CFS).

The Schirmer’s 1 test is a commonly used method to assess tear aqueous production by placing sterile filter paper strips along the inferolateral margin of the lower eyelid prior to any anesthesia. After placement, patients are instructed to gently close their eyes for five minutes, after which the strips are removed, and the extent of wetting is measured to evaluate tear secretion. Scores are recorded in millimeters and follow the scale below [36]. In a busy clinic setting, the rapid modified Schirmers 1 test is performed for one minute [37]. The scale is then multiplied by a factor of 3.5 to approximate the 5 min Schirmers 1 test [37].

**Figure 2:**
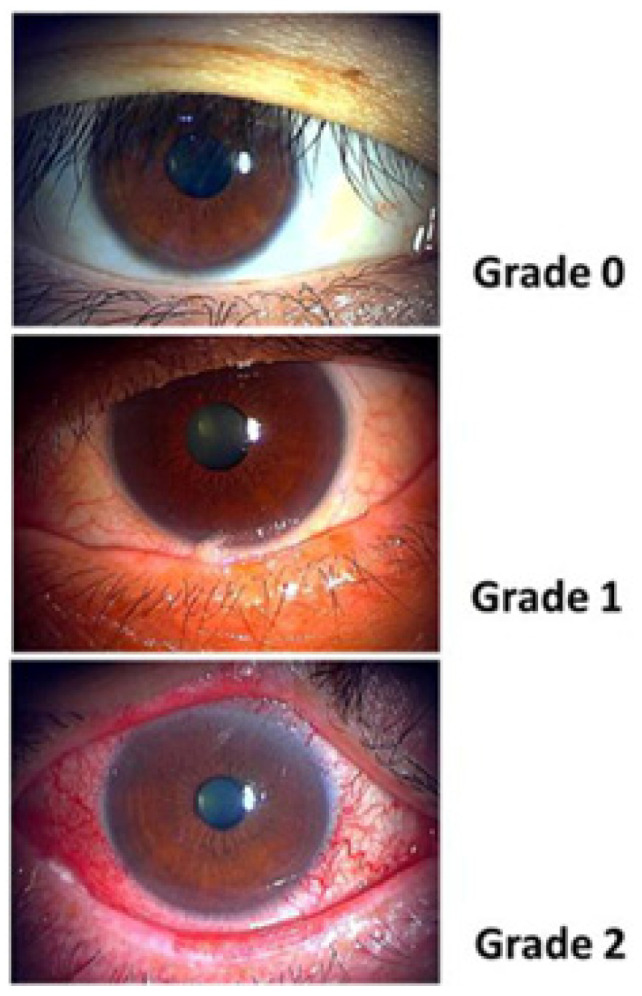
Clinical grading scale for conjunctival injection, where grades 0–2 indicate absent, mild, or moderate to severe redness, based on vessel dilation and hyperemia. Image from [25]. Copyright 2013 by Nature Publishing Group. Obtained with permission.

Conjunctival injection is the extent to which the blood vessels and the degree of redness of the bulbar conjunctiva is present [20,38]. The assessment is graded on a scale from 0 to 2, with higher scores indicating greater disease severity as seen in Figure 2. Palpebral conjunctival redness (PCR) and bulbar conjunctival redness (BCR) are valuable objective parameters that are good signs to follow ocular and lid margin surface inflammation. The BCR is currently the only parameter used when assessing the severity sum scores using the ICCGVHD criteria. However, BCR and PCR may often be discordant in oGVHD and it is important to consider both metrics.

**Figure 3:**
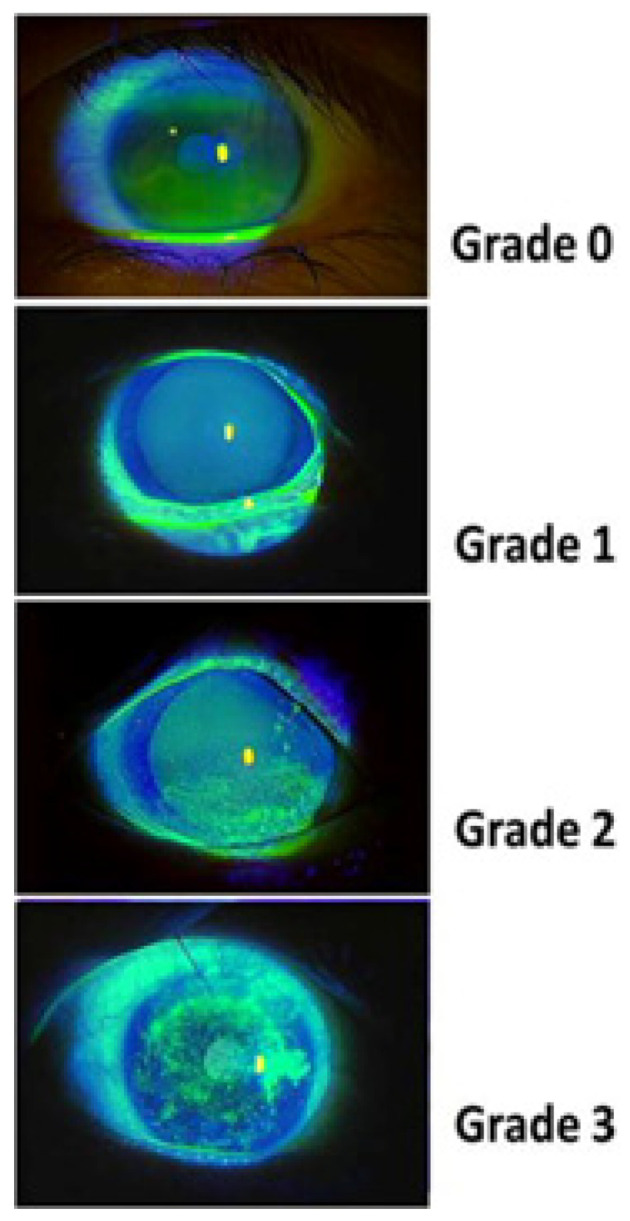
Grading scale for corneal fluorescein staining in oGVHD. Areas of epithelial damage are visualized using fluorescein dye under cobalt blue illumination. Severity is graded from 0 to 3 according to the extent and density of punctate epithelial erosions. Image from: [25]. Copyright 2013 by Nature Publishing Group. Obtained with permission.

Corneal fluorescein staining (CFS) is a key diagnostic tool in oGVHD. One drop of fluorescein dye is instilled into the patient’s eye and distributed by blinking, allowing the dye to coat the ocular surface. Then, with a cobalt blue light filter on the slit lamp, staining is easily visible. It is important to allow enough time for the fluorescein stain to reside over the ocular surface to penetrate the epithelial barrier to see more occult epithelial involvement. The CFS test assesses for epithelial cell damage, which is a hallmark indicator of disease activity [39]. Staining is graded on a 0–3 scale, with higher grades indicating greater underlying inflammation and associated epithelial damage [21,25]. The grading scale for CFS can be see in Figure 3.

The recorded values for each objective measure are combined into a composite score to determine both the diagnostic likelihood category, classified as definitive, probable, or none, and the overall severity score, which ranges from 0 to 12, with higher scores indicating more severe disease.

After the measurements for calculating likelihood of oGVHD are obtained, a full routine examination is performed to rule out alternative causes of the patient’s symptoms. This ensures that other ocular manifestations or comorbid conditions contributing to the patient’s symptoms are identified, since oGVHD is a broad clinical entity that can overlap with multiple causes of ocular surface disease [26,40].

#### 4.2.3. General Clinical Examination

One of the most important aspects of a patient’s reported symptoms is their visual acuity. Therefore, routine vision checks are performed in clinics to monitor whether vision is improving, stable, or worsening, as this reflects symptomatic change. If a patient does not reach 20/20, a pinhole test is performed. Improvement through the pinhole suggests the reduction is due to refractive error, whereas no improvement raises suspicion for underlying ocular pathology [41,42]. In oGVHD, visual acuity is often reduced due to ocular surface inflammation, tear film instability, and corneal epithelial damage, which disrupt optical quality [21,43].

A general eyelid examination is performed to assess findings such as dermatochalasis, lagophthalmos, snap-back lid laxity, and other lid abnormalities. These assessments are typically conducted without the need for a slit lamp or specialized ophthalmic equipment, allowing them to be completed quickly together during routine evaluation.

Early screening using non-invasive diagnostic techniques may further improve detection and reduce the risk of misdiagnosis in oGVHD. Adjunctive methods such as impression cytology can provide valuable information regarding epithelial integrity, goblet cell density, and ocular surface inflammation, often identifying subclinical disease before overt clinical findings become apparent [44].

Dermatochalasis refers to the redundancy and laxity of the eyelid skin and muscle, often referred to as “baggy eyes” [45,46]. Dermatochalasis can be assessed generally through the naked eye and is caused by normal aging processes that include things such as loss of elastic fibers, thinning of the epidermis, redundancy of the skin, and lymphatic dilation [47]. Excessive repair of the redundant tissue can also adversely impact ocular surface symptomatology.

Lagophthalmos is incomplete or abnormal closure of the eyelids [48]. The pathophysiology of lagophthalmos varies depending on the patient’s underlying condition. Clinical assessment is performed by directing light from a transilluminator upward beneath the upper eyelid to evaluate for any residual gap in eyelid closure. The lid separation is graded in millimeters. Any notable findings may adversely impact the success of surface treatments; usage of nighttime petrolatum ointments is often necessary.

Snap-back laxity refers to the lower eyelid’s ability to return to its original position after being displaced. The assessment is typically performed by gently pulling the lower eyelid downward, often with a cotton-tipped applicator, and holding it away from the globe for several seconds before release. The observed time required for the lid to return “snap back” to its natural position serves as the basis for grading the relative laxity and ability of the lid to oppose the ocular surface.

The degree of the lids’ ability to return to its more physiologic apposition reflects the ability to evenly spread the tear film and its components more evenly and completely over the ocular surface. Snap-back laxity is graded 0–IV (with IV representing severe laxity, or failure to return without a blink) [49]. Most of the laxity may be attributable to aging processes, but other etiologies of lower eyelid malposition, such as cicatricial conjunctival fibrosis, previous eyelid surgeries, trauma, cancer related radiation-chemo therapies and inflammatory processes, must be considered [50].

oGVHD can directly lead to lid abnormalities, so it is important that patients have a thorough eyelid examination to document any structural or functional changes. As part of the general assessment, visible eyelid abnormalities such as entropion, ectropion, blepharospasm, ptosis, eyelid margin scarring or irregularity, and lash misdirection should be carefully documented and optimize to a more normal anatomical and functional state. These findings are clinically important, as they may contribute directly to patient symptoms and provide useful clues in identifying the underlying cause. Considering and incorporating these lid margin observations into the overall ocular surface evaluation provides a more comprehensive diagnostic process and effective treatment of lid-related ocular surface associated comorbidities.

#### 4.2.4. Slit-Lamp Examination

Once the general external examination and basic objective assessments are completed, slit-lamp evaluation is performed. This examination allows for detailed assessment of the cornea, anterior chamber, iris, lens, and conjunctiva, anterior vitreous enabling the clinician to identify abnormal findings that may guide diagnosis and provide insight into the underlying cause of the patient’s symptoms. Photo slit lamp documentation particularly of corneal fluorescein staining, i.e., punctate staining for comparison after treatment will aid in serial follow up of this important objective surface diagnosis data point.

#### 4.2.5. Lid Margin and Meibomian Gland Assessment

After completing the slit-lamp evaluation, it is routine to perform a extensive lid margin assessment. This examination evaluates four key parameters: anterior blepharitis (AB), vascularity (V), obstruction of meibomian gland orifices (O), and turbidity of meibum secretions (T). This examination is referred to as the ABVOT lid margin exam.

**Figure 4:**
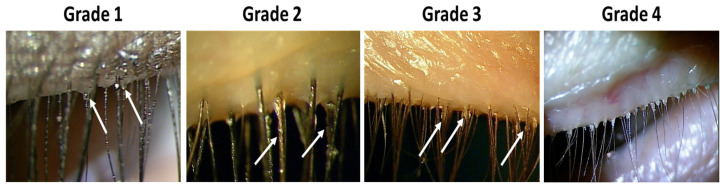
Slit-lamp images showing anterior blepharitis (AB) grades 1–4, reflecting progressive cylindrical dandruff and lid-margin inflammation.

Anterior blepharitis (AB) is assessed by examining the lash line for signs of inflammation and scaling or dandruff often manifesting as cylindrical or triangular sleeves around the eyelashes. This finding is frequently associated with Demodex infestation and reflects chronic lid margin irritation. The grading scale for this assessment can be seen in Figure 4.

These findings can be frequently associated with a history of recurrent styes, chalazion, and often seen with facial and ocular rosacea.

**Figure 5:**
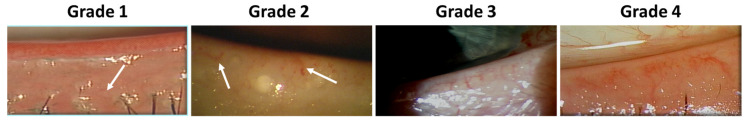
Meibomian gland changes in oGVHD. **Left** (Grade 1): normal appearance. **Right** (Grade 4): marked inflammation with orifice obstruction and vascular dilation.

Vascularity (V) refers to the presence of dilated or engorged blood vessels and telangiectasia at the lid margin. These vascular changes are indicative of persistent chronic inflammation and are commonly observed in oGVHD patients with long-standing ocular surface disease. The grading scale for this assessment can be seen in Figure 5.

Obstruction (O) is evaluated by applying adequate focal pressure along the lid margin, i.e., with a cotton tip applicator to determine whether the meibomian gland orifices are patent or blocked [51]. Evidence of obstruction, such as non-expressible glands, is a hallmark feature of meibomian gland dysfunction and directly contributes to evaporative dry eye. The grading system is based on the clinical assessment of relative meibum expressibility. A grade of 0 represents spontaneous easy meibum expression, while a grade of 4 indicates complete gland obstruction with no meibum secretion despite significant applied focal pressure [52]. It is important to remember that expression of meibum does not negate more distal obstruction of acini along the common duct of the meibomian gland apparatus. Any tenderness during expression can often suggest obstruction along the common duct.

**Figure 6:**
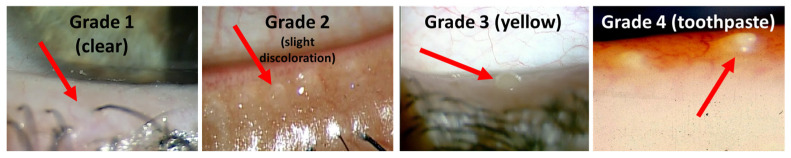
Meibum turbidity grading in meibomian gland dysfunction. **Left** (Grade 1): clear secretion with no obstruction. **Right** (Grade 4): opaque, turbid meibum indicating advanced gland dysfunction.

Turbidity (T) describes the quality of the meibum secreted from the glands. In healthy lids, the secretion is typically clear without debris, whereas in meibomian gland dysfunction, the meibum often appears cloudy, thickened, or toothpaste-like, reflecting abnormal glandular function and lipid processing [53,54]. Thick and turbid meibum may represent a systemic lipid abnormality and a lipid panel may be warranted [55]. The grading scale for this assessment can be seen in Figure 6.

Zone A is defined as the normally avascular region of the palpebral conjunctiva 0.5–1 mm posterior to the posterior lid margin [52]. Zone A vascular involvement is associated with chronic ocular surface irritation and inflammation, suggesting a variety of etiologies that promote vascular invasion.

Two additional objective ocular surface conjunctival eye and lid-associated assessments are bulbar conjunctival redness (BCR) and palpebral conjunctival redness (PCR). BCR and PCR refer to the relative redness of the bulbar and palpebral conjunctiva, respectively. Both BCR and PCR are graded on standardized scales from 10 to 100. A grade of 10 represents little to no observable hyperemic inflammation. As the grade increases up to 100, redness increases representative of severe inflammation. In non-inflamed eyes, BCR is typically clear. Normal PCR coloration may be observed as light salmon pink. The graded BCR scale was first presented by Schulze in 2007 [56], while the graded scale for PCR is still in the works of publishing. It has been notable that BCR may be distinctively independent from PCR in evaluating lid margin-associated ocular surface inflammation in oGVHD.

Together, the AB, V, O, T lid margin parameters, along with Zone A, BCR, and PCR, provide dynamic repeatable inflammation-related findings of eyelid and meibomian gland health. These findings are especially valuable in oGVHD because they can reveal early dysfunction that may not yet be apparent symptomatically. Clinicians may use these parameters as an important tool for correlating lid pathology with patient complaints of ocular surface discomfort and dryness.

Meibomian glands are frequently affected in oGVHD, making evaluation of their structure a critical part of the diagnostic process. Evaluation of meibomian gland structure and function prior to SCT should always be routinely performed whenever possible. One might even consider obtaining a baseline exam when the patient is first diagnosed with any hematologic malignancies because the status of these glands will change over the course of hematologic cancer treatments, which may have its etiology from a variety of factors including aging, chemotherapy, SCT.

**Figure 7:**
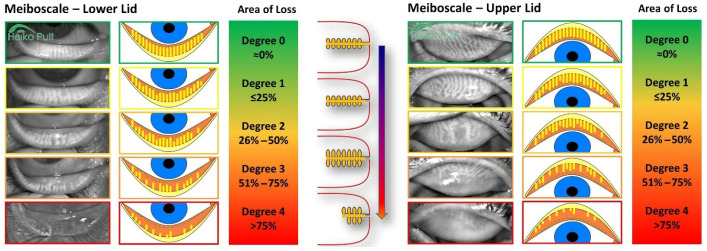
Meiboscale measuring areas of glandular dropout. Degree of meibomian gland loss is graded 0 (no loss; 0% area lost) to 4 (significant loss; greater than 75% area lost). Image from: [57].

Meibography is the preferred method of evaluating gland structure and involves eversion and infrared visualization of the upper lid (most commonly). This technique provides a clear view of gland morphology and highlights areas of dropout that may not be visible on routine slit-lamp examination. The degree of gland loss is often quantified on a grading system, which ranges from 0 to 4 and provides standardized documentation of glandular dropout. A Meibo-score of 0 represents normal gland anatomy; the higher the score, higher areas of meibomian gland dropout and truncation are noted. This scale allows clinicians to categorize the extent of structural dropout and compare disease severity across patients (Figure 7).

If a meibographer is unavailable, the lower lid can be transilluminated at the slit lamp using a muscle light after turning off the slit lamp beam. This simple technique allows assessment of meibomian gland anatomy and atrophy by evaluating the presence or absence of gland silhouettes on transillumination.

#### 4.2.6. Intraocular Pressure

Measurement of intraocular pressure (IOP) is an essential part of the evaluation, as cases of ocular hypertension and secondary glaucoma have been reported in patients with oGVHD [1]. The iCare IC100 tonometer is commonly used in clinical practice to obtain rapid, reliable, non-invasive pressure measurements. Measuring the IOP with this device prior to surface examination does not impact the corneal assessment unlike the Zeiss applanation tonometer. Recording IOP as well as any family history of glaucoma provides important context for risk stratification and long-term monitoring.

#### 4.2.7. Dilated Fundus Examination

It is essential to perform a dilated fundus examination to evaluate for potential posterior segment involvement. Although less common than anterior segment disease, posterior segment complications have been reported in GVHD, including microvascular retinopathy characterized by cotton-wool spots, intraretinal hemorrhages, and occasional vitreous hemorrhage, which may be related to the underlying disease, conditioning regimens, or immunosuppressive therapy [58]. Additionally, central serous chorioretinopathy (CSCR) has been observed in hematopoietic stem cell transplant patients [59]. Posterior scleritis and vitritis, while uncommon, have also been described and may reflect underlying inflammatory involvement of the posterior segment [60,61]. Regular dilated fundus examinations are therefore important for early detection and management of these potentially sight-threatening complications.

### 4.3. Importance for Comprehensive Examination

This completes the routine examination that is essential for every patient, but it carries particular significance in those with oGVHD. Given the wide spectrum of ocular manifestations observed in this disease, it is critical to examine all aspects of the eye in a systematic and comprehensive manner. Standard assessments such as the Schirmer’s 1 test, PCR/BCR grading, and corneal fluorescein staining are part of routine ophthalmic practice, but in oGVHD they are indispensable as they form the foundation of the diagnostic criteria. In addition to these core evaluations, more recent studies have emphasized the role of advanced imaging modalities, including tear film interferometry and in vivo confocal microscopy, which allow for detailed assessment of tear film dynamics and corneal microstructural changes [24].

**Figure 8:**
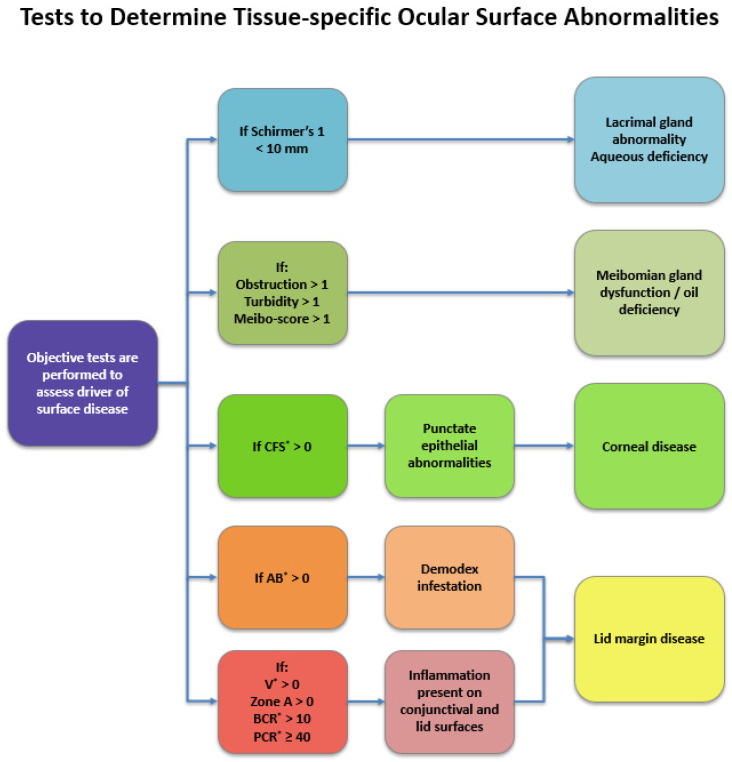
Workflow for evaluating ocular surface abnormalities. The flowchart shows tests conducted to assess and differentiate tear, oil, and inflammation drivers of surface disease. Schematic created by authors. * See abbreviations list on page 25.

By performing this thorough evaluation, clinicians can more precisely determine which ocular manifestations are driving a patient’s symptoms and, in turn, lay the groundwork for informed treatment decisions. This progression from diagnosis to intervention highlights the importance of transitioning into a structured management approach allowing serial follow-up examinations addressing the ocular surface anatomy and the major surface-related components, i.e., water, oil, and inflammation. A simplified schematic outlining the diagnostic workflow is presented in Figure 8.

### 4.4. Current Management of Ocular GVHD at MDACC

The foundation of oGVHD treatment lies in recognizing that acute conditions require acute interventions, whereas chronic diseases demand long-term, chronic treatment. It is essential to convey to patients from the outset that oGVHD is a chronic condition that must be managed over time. The primary goals of therapy are to alleviate symptoms, preserve vision, and reduce disease burden on quality of life.

Treatment is tailored to the specific ocular surface issues causing the patient’s discomfort. This approach addresses each abnormal finding based on the patient’s symptoms and clinical signs. It is common for signs and symptoms not to correlate perfectly, so both should be considered whenever possible. Key findings may include poor tear quality, reduced tear volume, or decrease reflex tearing due to lacrimal gland fibrosis. Additionally, the cornea, conjunctiva, meibomian glands, and lacrimal glands should be evaluated. By systematically targeting each affected component, the overall disease burden can be reduced, helping patients achieve better symptom control, visual acuity, and quality of life.

#### 4.4.1. Management of Lacrimal Gland Dysfunction

Patients with oGVHD often present with symptoms of ocular surface dryness, irritation, and discomfort when lacrimal gland dysfunction develops as a secondary manifestation of the disease [59]. A key diagnostic objective measurement is the Schirmer’s 1 test, with scores less than 10 mm indicating dryness. While many dry eye offices do not perform Schirmer’s testing, we find it useful to define the severely aqueous deficient oGVHD patient who with multiple consecutive testing cannot produce an adequate volume. To address these symptoms and to reduce the burden of oGVHD, several therapeutic approaches are available, each aimed at improving tear production, tear volume or optimizing tear quality to effectively stabilize the ocular surface and enhancing patient comfort.

##### Tear Supplements

Artificial tears have long been considered the first-line treatment for lacrimal gland dysfunction (LGD), as they are simple, cost-effective, and provide efficient relief for mild or sudden dry eye symptoms [62]. However, while artificial tears help alleviate discomfort, their benefit is temporary, as they do not address the underlying pathology of LGD [62]. Over the years, the production of artificial tears has expanded into hundreds of formulations, each differing in composition. Early products containing preservatives gained popularity for their extended shelf-life, but their use declined after reports of stinging and long-term ocular surface toxicity [62]. As a result, preservative-free formulations of artificial tears are preferred [63].

At MDACC, artificial tears can be a monotherapy for oGVHD or are considered and incorporated as an adjunct to other therapies, as they provide supportive relief and contribute to an overall synergistic benefit. Routine regular dosing regardless of symptoms are encouraged especially when repeated Schirmer’s 1 suggests low aqueous volume and an inability to provide reflex tearing.

##### Punctal Occlusion

One of the most common strategies for improving tear retention and aqueous volume is punctal occlusion. The punctal openings function as the primary outflow pathway for tears. By occluding the punctal openings, tear drainage is reduced, which results in greater tear retention on the ocular surface. Punctal occlusion is particularly recommended for patients with Schirmer’s 1 scores below 10 mm who consistently report significant ocular dryness. Approaches to punctal occlusion include cautery, plugging, and injection of high molecular weight sodium hyluronate gel.

One approach to punctal occlusion involves the use of silicone punctal plugs, which are available in different sizes to accommodate individual punctal anatomy. Their primary advantage lies in their reversibility. If epiphora develops, the plugs can be easily removed. However, although they are simple to insert, they are prone to extrusion, limiting their long-term effectiveness. One report showed that the majority of oGVHD patients who received punctal plugs experienced plug loss within a 90-day period [64]. Additionally, other adverse effects of punctal plugs include foreign body sensation, infection, irritation, and granuloma development. Temporary collagen plug inserts are not usually recommended. Once inserted, it is difficult to know if occlusion is still present once the collagen insert has been added.

Due to the adverse effect profile and limited long-term efficacy of punctal plugs and collagen inserts, cautery as the initial approach is preferred at MDACC. With fine tip radiofrequency electrocautery, the punctum is closed through scar formation that is induced by thermal energy to produce a delicate very thin-walled scar membrane over the punctal opening. The major advantage of cautery is durability, as the closure often persists until the punctum spontaneously reopens, which can vary considerably between patients [65]. The limitation of this approach is its irreversibility in the short term if excessive tearing occurs, the closure cannot be easily undone, and one must wait for the punctum to naturally reopen or undergo surgical intervention. Additionally, patients may be reluctant and apprehensive to any surgical procedure, and the relative irreversibility leads many patients to prefer plugging. Despite this, punctal cautery remains a valuable option in patients with severe or refractory disease where sustained occlusion is required.

Lastly, once cautery is performed for one of the two punctal openings, the other opening is purposedly plugged with a silicone plug on the same side to ensure the closure does not induce an unwanted uncontrolled epiphora.

Injection-based methods of punctal occlusion represent an intermediate option.when hyaluronic acid canalicular gels are injected with a cannula into the puncta. Importantly, patients are instructed not to disturb the area or sneeze after the procedure, which may result in displacement of the gel. This method is not dependent on punctal size, since the gel adapts to and fills the canalicular space. Thus, injections are an appropriate option for punctal occlusion before proceeding to plugs or cautery.

##### Lacrimal Gland Stimulation

Lacrimal gland stimulation with oral muscarinic agonists such as pilocarpine (Salagen) and cevimeline represents another strategy for managing lacrimal gland dysfunction. These agents act on muscarinic receptors of the lacrimal gland epithelium to enhance tear secretion. Despite their therapeutic potential, tolerability remains a significant challenge: for example, one study reported that 96.8% of patients receiving pilocarpine experienced systemic side effects such as sweating, nausea, dizziness, and visual disturbances, largely attributable to off-target parasympathetic activation [66]. Further, stimulation of the lacrimal gland when it is itself diseased may have limited yield in tear production.

Another method of tear stimulation involves the use of nasal sprays such as varenicline (Tyrvaya) Tyrvaya increases tear secretion via nasolacrimal reflex and has demonstrated clinical efficacy in patients with oGVHD [67]. However, long-term safety and effectiveness remain to be fully established in these patients.

In addition to pharmacologic treatments, device-based therapies have been developed to stimulate the lacrimal gland. One such device, the iTear100, applies external neural stimulation to the nasal bridge to trigger basal tear secretion [68]. While early studies did not demonstrate consistent long-term symptom relief, the device has been shown in several reports to increase tear production and reduce dryness symptoms [68].

##### Autologous Serum Eye Drops

Autologous serum eye drops (ASEDs) represent a valuable therapeutic option for patients with oGVHD. Because they are formulated from the patient’s own serum, these drops provide growth factors and anti-inflammatory mediators that closely mimic the composition of natural tears, thereby promoting ocular surface healing [30]. Clinical studies have demonstrated that patients with severe, refractory dry eye secondary to oGVHD treated with ASEDs experience improved corneal sensitivity along with reduction in both the frequency and severity of symptoms [69]. In light of their proven effectiveness, autologous serum eye drops are considered a key component of clinical management and are commonly employed to treat lacrimal gland dysfunction alongside the broader spectrum of ocular graft-versus-host disease.

##### Platelet-Rich Plasma Eye Drops

Platelet-rich plasma (PRP) drops is an emerging autologous biologic therapy for severe ocular surface disease and has been proposed as an alternative to autologous serum eye drops [69]. Clinical evidence demonstrates that PRP eye drops significantly improve corneal fluorescein staining, TBUT, and patient-reported symptoms in severe dry eye refractory to conventional therapy [70]. Additional studies have shown that PRP treatment is associated with reduction in ocular surface inflammation and improvements in conjunctival and corneal parameters [71]. PRP has been shown to contain higher concentrations of epithelial growth factors compared with autologous serum, suggesting it may be more suitable in treatment of corneal epithelial defects [70]. However, to date no study comparing efficacy of PRP vs. autologous serum has been performed in oGVHD. In this context, platelet-rich plasma eye drops may be particularly beneficial in patients with predominant corneal epithelial defects, whereas autologous serum eye drops remain a more suitable overall option for managing the broader spectrum of ocular graft-versus-host disease manifestations [72,73].

#### 4.4.2. Management of Conjunctival Dysfunction in oGVHD

Patients with oGVHD variably present with conjunctival involvement, which may manifest as hyperemia, chemosis, conjunctival abrasions, pseudomembrane formation, conjunctivitis, cicatricial changes, conjunctivochalasis, symblepharon and severe dry eye symptoms [1,40]. Objective findings in these patients often include elevated palpebral/bulbar conjunctival redness (PCR/BCR) scores, conjunctival staining defects, folds in the conjunctiva or limbal region consistent with conjunctivochalasis, and other characteristic changes depending on the specific type and severity of conjunctival disease present.

##### Topical Glucocorticoids

Topical glucocorticoids, such as 1% prednisolone acetate, are frequently employed in patients with oGVHD for their potent anti-inflammatory effects and their ability to promote lymphocyte apoptosis. Clinical studies have demonstrated symptomatic and objective improvement with prednisolone acetate and other low-dose steroid formulations [16,74]. However, the prolonged use of topical corticosteroids carries well-recognized risks, including elevated intraocular pressure, cataract formation, corneal thinning, and an increased susceptibility to infectious keratitis [75,76]. Thus, while glucocorticoid therapy is commonly employed as a mainstay of treatment for exacerbations or complications of oGVHD as they arise, there remains a need for long-term, chronic treatment of oGVHD. A preservative free topical formulation may be necessary when treating long-term is necessary.

##### Lifitegrast (Xiidra)

Lifitegrast ophthalmic solution 5% (Xiidra) is an LFA-1 integrin antagonist commonly used in dry eye disease and has been investigated as a potential steroid-sparing therapy for oGVHD-related dry eye. In a randomized, double-blind trial of 32 patients with diagnosed oGVHD, lifitegrast significantly reduced symptom severity as measured by the Symptom Assessment iN Dry Eye (SANDE) score and improved Schirmer’s 1 test results compared to placebo after four weeks of treatment [77].

Although improvements in other endpoints such as OSDI, tear film breakup time, and corneal fluorescein staining did not reach statistical significance, the treatment demonstrated a favorable safety profile, with no adverse effects on visual acuity or intraocular pressure. These findings support lifitegrast’s role as a safe adjunctive option for alleviating ocular surface inflammation in oGVHD, while showing the need for larger and longer-term studies to confirm its efficacy in this patient population [77].

##### Topical Calcineurin Inhibitors

The application of topical calcineurin inhibitors to the ocular surface has produced variable outcomes, with the two primary agents being cyclosporine A (CSA) and tacrolimus. CSA has demonstrated clinical benefit in some oGVHD patients, particularly those who fail to respond adequately to other therapies [78,79]. Topical CSA has been shown to improve lacrimal gland secretion as well as goblet cell densities. However, overall therapeutic effect of topical calcineurin inhibitors is often limited by modest efficacy and poor tolerability [79]. Many patients continue to experience significant symptoms despite treatment, and objective measures such as tear production frequently show minimal or statistically insignificant improvement, even with frequent dosing [25]. Up to one-third of patients ultimately discontinue CSA due to adverse events including burning sensations, redness, and other ocular symptoms [80,81]. Symptomatic improvement in approximately 62.5% of patients with conjunctival involvement has been demonstrated [20].

Topical tacrolimus has been shown to be safe, generally well tolerated, and effective in the management of oGVHD [82,83]. Topical tacrolimus represents a valuable alternative for patients who are unable to tolerate CSA [82]. While both agents provide comparable therapeutic benefits, their use highlights the importance of calcineurin inhibition as a steroid-sparing strategy in controlling ocular surface inflammation.

##### NET-Targeted Therapies

The primary agent investigated for NET-targeted therapy in oGVHD is topical heparin, which is thought to reduce neutrophil extracellular trap (NET) proteins and modulate fibrosis and inflammation. At sub-anticoagulant concentrations (100 IU/mL), heparin has demonstrated potential in decreasing NET-associated conjunctival inflammation. However, topical heparin usage may increase the risk of corneal stromal or subconjunctival hemorrhage, and its precise mechanism of action along with long-term safety profile remain under investigation [20]. However, topical heparin is seen as a good non-steroidal option to control inflammation when patients are not responsive to other treatments. Heparin is often used adjunctively with topical spironolactone at MDACC to treat recalcitrant punctate epithelial keratopathy. Controlled clinical trials are needed to demonstrate efficacy.

##### Topical Dapsone

Topical dapsone inhibits neutrophil recruitment and reduces oxidative damage to the ocular surface, which uniquely positions it as a potential adjunctive agent in oGVHD [84]. Dapsone is well suited for cases of marked conjunctival inflammation involvement in oGVHD, especially when conventional treatments such as steroids, calcineurin inhibitors and topical spironolactone are not sufficient alone to reduce bulbar and palpebral hyperemia. However, since dapsone is a sulfa-based derivative, it may be contraindicated in patients with sulfa allergies. Further clinical studies are needed to establish its safety, efficacy, and long-term impact in this patient population.

##### Topical Methotrexate

Topical methotrexate (MTX) may be an additional therapeutic option for controlling and reducing ocular surface inflammation. A 2025 study demonstrated that topical MTX dosed at 1 mg/mL four times daily not only shows minimal cytotoxicity in preclinical models but also was associated with decreased palpebral conjunctival hyperemia, improved OSDI scores, and improved corneal fluorescein staining (CFS) within a 2-week treatment period [85]. Improvements in Schirmer’s 1 and tear-film stability, although not statistically significant, suggest that MTX may be beneficial in the management of recalcitrant inflammation-driven ocular surface disease.

##### Other Miscellaneous Therapies

Topical application of 1% progesterone gel to the forehead has been used in oGVHD patients and has been associated with an improvement in overall ocular symptoms. However, because the site of application does not have a plausible explanation for the improvement in symptomatology, topical progesterone to the forehead is not widely regarded as a first-line therapy [86].

Systemic gentamicin has also been described in treatment of oGVHD, with observed benefits including suppression of inflammatory cell infiltration and fibrosis in cGVHD organs. Despite these clinical benefits, systemic administration of gentamicin is generally avoided in oGVHD patients due to the risk of interactions with concurrent systemic medications and the potential for systemic toxicity [87]. Epithelial toxicity from topical gentamycin is well documented and is not the antibiotic of choice in ocular surface diseases [88]. Thus, the risk–benefit profile of gentamicin in oGVHD is unclear.

#### 4.4.3. Management of Meibomian Gland Dysfunction in oGVHD

Meibomian gland dysfunction (MGD) in oGVHD is characterized by gland dropout, reduced or abnormal meibum secretion, and significant alterations in the lipid layer of the tear film based on abnormal TBUT. Symptomatically, patients present with classic dry eye complaints due to tear film instability, while clinical findings often include decreased TBUT, increased gland dropout, poor gland expressibility or obstruction from distal or proximal ductal fibrosis and altered meibum quality. Advanced MG atrophy is common in oGVHD and is associated with lipid insufficiency in the tear film. Advanced cases of lipid insufficiency leading to evaporative dry eye may present with punctate epithelial erosions, conjunctival and corneal inflammation, and other secondary ocular surface changes. General treatment paradigms of MGD in oGVHD do not differ significantly from primary MGD and focus on either improving meibum obstruction/production or supplement the lipid component of the tear film directly.

##### Conventional MGD Therapies

These conventional therapies are non-pharmacologic approaches, relying on external methods to support the ocular surface. Warm compresses and external heat masks form a traditional cornerstone in the management of MGD. Clinical studies have shown that patients treated with these approaches experience significant improvement in both subjective ocular symptoms and objective clinical signs [89]. However, treatment adherence is often a limitation due to difficulties with easy access to hot water as well as the tedious nature of the routine. Moreover, female patients often complain about warm compresses because of cosmetic concerns, i.e., smearing the patient’s make-up.

Another commonly used adjunct therapy at the oGVHD service at MDA is blinking exercises followed with saline rinsing of the ocular surface. This patient-initiated approach is recommended instead of warm compresses. Patients are asked to use 0.9% sodium chloride saline nebulizer solution for ocular use. Instructions are as follows: firmly blink nine times in succession to mechanically express meibum from the glands onto the ocular surface and repeat multiple times a day. After the consecutive blinks are completed, the patient instills multiple drops of 0.9% sodium chloride used for a vehicle for nebulization as the preservative free saline to flush away any secretions and debris expelled by the forced blinking. Furthermore, any toxic accumulations the patients may be sensitive to are diluted. This process helps reduce the impact of abnormal lipid secretions on the tear film, improve any gland obstruction, and alleviate general symptoms of ocular discomfort. The regimen can be performed initially three times daily and can be modified based on patient compliance.

##### Perfluorohexyloctane (Miebo)

Miebo is a novel ophthalmic solution widely used for the treatment of dry eye disease (DED), and it has become particularly valuable in patients with oGVHD who present with Meibomian gland dysfunction (MGD). As a single-entity, water-free, steroid-free, and preservative-free semi fluorinated alkane, Miebo works by stabilizing the tear film and reducing tear evaporation caused by evaporative dry eye [90]. Clinical studies have shown significant improvements in both the signs and symptoms of DED, with the drug generally well tolerated [91]. Common adverse events associated with the use of Miebo include vitreous detachment, allergic conjunctivitis, blurred vision, and increased lacrimation [92]. No clinical trials in oGVHD are currently available.

##### Topical Antibiotic Ointments

Topical antibiotics such as topical 1% azithromycin and erythromycin offer a valuable but often underutilized approach to the management of MGD [93,94]. Azithromycin exerts both antimicrobial and anti-inflammatory effects, reducing lid margin bacterial colonization, suppressing pro-inflammatory cytokines, and improving the quality and expressibility of meibum [93]. Clinical studies have demonstrated that topical azithromycin is associated with significant improvements in symptoms, ocular surface signs, and tear film stability [94]. Notably, topical azithromycin has been shown to be associated with greater short-term improvement in tear film quality compared to oral azithromycin or doxycycline [93].

##### Topical Spironolactone in MGD

Topical spironolactone, a mineralocorticoid aldosterone receptor antagonist, has emerged as a promising adjunctive therapy for ocular graft-versus-host disease, particularly in patients with meibomian gland dysfunction (MGD) [95]. Beyond its established systemic use, topical spironolactone has demonstrated anti-inflammatory and antifibrotic effects that may be beneficial in modulating oGVHD-related ocular surface inflammation and glandular fibrosis [95,96]. Clinical and early observational studies have reported improvements in lid margin vascularity, meibum quality, corneal fluorescein staining, tear film stability, and patient-reported symptom scores, with favorable tolerability and adverse effects generally limited to mild transient stinging [95,96,97]. Unlike topical glucocorticoids, spironolactone does not carry the risks of elevated intraocular pressure, cataract formation, delayed epithelial healing, or increased infection risk, making it an attractive steroid-sparing option for chronic disease management [95,98,99]. Current evidence remains limited to small clinical series and early-phase studies. Larger, controlled trials are needed to more clearly define its efficacy, optimal dosing, and long-term safety, as well as to determine its therapeutic role across the broader spectrum of oGVHD-related ocular surface involvement beyond meibomian gland dysfunction.

##### Intense Pulse Light (IPL) Therapy

Intense pulsed light (IPL) therapy is a novel treatment in which controlled pulses of light are applied to the lower eyelids to liquefy inspissated meibum and improve gland function. Also, IPL has been shown to modulate extracellular matrix remodeling by regulating matrix metalloproteinase-1 (MMP-1) and transforming growth factor-β1 (TGF-β1) secretion in fibroblasts, suggesting a role in collagen remodeling through MAPK signaling pathways. The role of IPL in management of meibomian and lid margin disease has been demonstrated with improvements in subjective symptoms and tear film stability observed in multiple studies [100]. Prospective controlled clinical trials in oGVHD are currently lacking.

#### 4.4.4. Management of Corneal Dysfunction in oGVHD

Corneal involvement in oGVHD encompasses a broad spectrum of disease, making symptom-based diagnosis difficult. Patients may present with nonspecific complaints, while objective findings often include elevated CFS scores, punctate epithelial keratopathy, and filamentary keratitis on slit-lamp examination. In more severe cases, progressive changes such as corneal erosions, stromal thinning, ulceration, or even perforation may be observed [21]. Several therapies for corneal dysfunction in oGVHD are described below:

##### Amniotic Membrane

Amniotic membrane transplantation (AMT) has demonstrated considerable utility in treating corneal complications associated with oGVHD, particularly when standard therapies fall short. In one case, multi-layer AMT successfully stabilized a calcareous corneal degeneration and perforation and preserved ocular surface integrity for over 20 months [101]. In a separate instance, self-retained sheet AM (ProKera) rapidly re-epithelialized persistent erosions and reduced inflammation, even as conventional treatments failed [102]. More recently, AMT effectively managed a descemetocele by restoring corneal thickness and providing symptomatic relief while awaiting further surgical options [103]. Overall, AMT remains a reliable option for patients with persistent corneal defects, offering both objective improvement and symptomatic relief when conventional therapies prove inadequate.

##### Scleral Lens

The use of scleral lenses is a well-described approach for managing ocular surface and corneal complications in oGVHD. Scleral lenses are particularly effective in patients with severe dry eye disease, as they vault over the cornea and create a tear-filled reservoir that continuously hydrates and protects the ocular surface. Although randomized head-to-head trials comparing scleral lenses with standard lubrication therapy in oGVHD are lacking, observational studies have demonstrated their efficacy not only in treating corneal dysfunction but also in alleviating broader ocular symptoms in oGVHD, with improvements reported in visual acuity, OSDI scores, and corneal fluorescein staining [104]. A survey of 723 eye care providers demonstrated that scleral lenses were ranked as the second most preferred option for ocular surface disease management, following rigid gas-permeable corneal lenses [105].

##### Albumin Eye Drops

Albumin eye drops show therapeutic potential in managing corneal surface disease and refractory epithelial defects. Studies have shown that albumin accelerates epithelial healing in animal models and improves corneal staining scores in severe dry eye cases [106]. In oGVHD, albumin drops provided symptom relief in over 90% of patients with substantial clinical improvements [107]. Albumin eye drops are a promising adjunctive therapy for corneal epithelial healing and severe dry eye disease, particularly in cases resistant to standard treatment. Further, albumin eye drops are a much more accessible adjunct treatment than autologous serum or platelet-rich plasma.

#### 4.4.5. Ivermectin Ointment in Demodex Management

Demodex infestation often presents as anterior blepharitis and is commonly referred to as ‘eyelash mites.’ These microscopic organisms live within the lash follicles and sebaceous glands, and in cases of overgrowth, can trigger ocular symptoms such as irritation, redness, and eyelid inflammation. Patients may also report a history of flaking or itching around the follicular regions, including the eyelashes and sometimes the scalp. A history of recurrent styes and chalazion is often reported and maybe comorbidities creating meibomian gland dysfunction and atrophy.

The standard treatment of Demodex blepharitis at MDACC is the application of ivermectin ointment to the eyelashes nightly for two weeks. This regimen has been shown to be effective in reducing mite burden and improving associated eyelid inflammation and patient symptoms [108]. Treatment can be repeated as needed if cylindrical sleeves are recurring. Systemic treatment can be considered for more severe recurring recalcitrant lid or skin involvement. It is common to see facial skin involvement with ocular involvement.

In patients with a recent history of scalp or eyebrow flaking, tea tree oil shampoos are also commonly prescribed. These products are used to limit reinfestation from adjacent areas and have been shown to improve mite counts and eyelid findings [109].

## 5. Future Directions

Ocular GVHD treatment is advancing, but major gaps still limit improved outcomes including inconsistent diagnostic criteria, poorly understood treatment pathways, and limited evidence base trials for newer effective therapies.

A unified, ophthalmology diagnostic standard is necessary, utilizing standardized symptom scales like the OSDI and semi-objective tests such as the Schirmer’s 1, PCR/BCR, corneal fluorescein staining, conjunctival hyperemia and eyelid margin assessments including ABVOT and meibography is important in achieving uniform and repeatable assessment of disease activity. Recent single-cell RNA sequencing studies of the ocular surface have shown that high resolution cellular profiling can identify disease specific cell populations, transcriptional signatures, and molecular pathways involved in ocular surface inflammation and fibrosis. These findings support the potential development of biomarker-driven and precision-based diagnostic frameworks for oGVHD [110]. Artificial intelligence-driven digital tools like automated image analysis and symptom diaries can help close the gap between symptoms and signs to support a responsive, tissue targeted approach.

Of note, the above-mentioned diagnostic and therapeutic approach was developed contemporaneously with the TFOS DEWS II report, to which multiple citations are made throughout this work. The TFOS DEWS III report reflects the increasing recognition of dry eye disease as a multifactorial clinical entity with pathology not only of the tear film but also of the ocular surface and adnexae, reflecting recognition of both anatomic and physiologic factors as drivers of dry eye disease [111]. Further discussion on the many treatments entailed therein would be outside the scope of this work. However, it should be recognized that current treatment paradigms of oGVHD and dry eye disease have trended toward recognizing underlying drivers of pathology and tailoring personalized therapies rather than adopting standardized severity-based therapies in the past several years.

**Figure 9:**
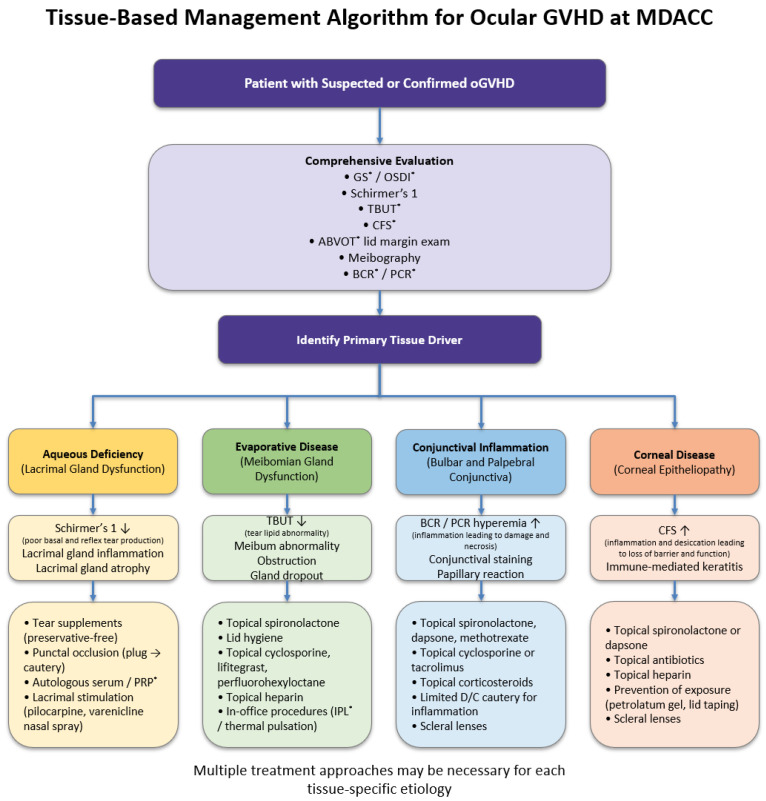
Tissue-Based Management Algorithm for Ocular Graft-Versus-Host Disease at MDACC. Schematic overview of the MDACC phenotype-targeted approach to oGVHD. Schematic created by authors. * See abbreviations list on page 25.

Management of oGVHD should focus on tissue target specific treatments in hopes of restoring and optimizing the ocular surface phenotype and its function. Aqueous-deficiency disease may be addressed with tears, punctal occlusion, platelet derived growth factor injection, drops or tear stimulation pharmacologically or mechanically; evaporative disease with lid margin hygiene, heat, azithromycin, or IPL; conjunctival disease with steroid-sparing anti-inflammatories; and corneal-predominant disease with serum drops, amniotic membrane, or scleral lenses. Further study of the role and durability of emerging agents and devices such as spironolactone, methotrexate, dapsone, NET-modulators, perfluorohexyloctane, IPL, and neuromodulatory stimulators are warranted. Multicenter, masked prospective trials using standardized endpoints are undoubtedly needed. A schematic overview of the MDACC phenotype-targeted management approach is shown in Figure 9.

Moreover safety, accessibility efficacy and affordability should be built into every treatment pathway. Priorities include long-term monitoring of adverse effects, i.e., steroid-related cataract and IOP risk, systematic tracking of retinal and posterior segment events, and pharmacovigilance for novel topical drugs. Studies should address cost, adherence, and efficacy as the treatments’ accessibility may vary widely by geography, socioeconomic factors, and dosing. Tele-ophthalmology, home monitoring of IOP and vision, and standardized nurse-led protocols for medication reconciliation and symptom tracking can expand access and promote follow-up remotely from major cancer centers. Lastly, an oGVHD registry linking the disciplines of hematology-oncology and ophthalmology is needed. Such a data sharing registry should include transplant variables, ocular phenotypes, treatments, and outcomes to enable further study of predictors of visual outcome, ocular phenotypes, and efficacy of phenotype-based approaches to treatment of oGVHD as previously discussed in this work. Understanding epidemiology and real-world effectiveness of phenotype-driven treatment approaches is one cornerstone of bringing oGVHD care into the era of precision medicine.

However, at present these attempts are nascent and highlight the need for inter-institutional and international data sharing to guide further frameworks for treatment and diagnosis. Until consensus guidelines are in place, the MDACC phenotype-targeted approach remains a practical framework of measuring carefully, treating the underlying driver, escalating rationally, and tracking outcomes objectively.

## Data Availability

Data sharing is not applicable to this article as no new data were created or analyzed in this study.

## Abbreviations

The following abbreviations are used in this manuscript:

AB	anterior blepharitis
ABVOT	anterior blepharitis, vascularity, obstruction, turbidity
aGVHD	acute graft-versus-host disease
allo HSCT	allogeneic hematopoietic stem cell transplantation
AM	amniotic membrane
AMT	amniotic membrane transplantation
AT1R	angiotensin II type 1 receptor
BCR	bulbar conjunctival redness
CFS	corneal fluorescein staining
cGVHD	chronic graft-versus-host disease
CSCR	central serous chorioretinopathy
CSA	cyclosporine A
DED	dry eye disease
FBS	foreign body sensation
GS	global symptom score
GVHD	graft-versus-host disease
HLA	human leukocyte antigen
HSCT	hematopoietic stem cell transplantation
ICCGVHD	International Chronic Ocular Graft vs. Host Disease Consensus Group
ICM	International Consensus Meeting
IOP	intraocular pressure
IPL	intense pulsed light
KCS	keratoconjunctivitis sicca
LGD	lacrimal gland dysfunction
MGD	meibomian gland dysfunction
MMPs	matrix metalloproteinases
MTX	methotrexate
MDACC	MD Anderson Cancer Center
NET	neutrophil extracellular trap
NIH	National Institutes of Health
oGVHD	ocular graft-versus-host disease
OSDI	Ocular Surface Disease Index
PCR	palpebral conjunctival redness
PEDs	persistent epithelial defects
PRP	Platelet-rich plasma
QoL	quality of life
RAS	renin angiotensin system
SCT	stem cell transplantation
SANDE	Symptom Assessment in Dry Eye
TBUT	tear breakup time
TFBUT	tear film breakup time
TFOS DEWS II	Tear Film and Ocular Surface Society Dry Eye Workshop II
TNFα	tumor necrosis factor
